# Complete mitochondrial genome of the pumpkin fruit fly, *Bactrocera depressa* (Diptera: Tephritidae)

**DOI:** 10.1080/23802359.2017.1285212

**Published:** 2017-02-06

**Authors:** Su Yeon Jeong, Min Jee Kim, Jong Seok Kim, Iksoo Kim

**Affiliations:** College of Agriculture & Life Sciences, Chonnam National University, Gwangju, Republic of Korea

**Keywords:** Mitochondrial genome, *Bactrocera depressa*, pumpkin fruit fly

## Abstract

In this study, we sequenced the complete mitochondrial genome (mitogenome) of the pumpkin fruit fly, *Bactrocera depressa* (Diptera: Tephritidae), which is an economically damaging pest of pumpkin and turban squash. The 15,832-bp-long complete mitogenome of the species consists of a typical set of genes, with an arrangement typical of insects. Of the 13 protein-coding genes (PCGs), 12 have a typical ATN start codon, whereas the *COI* gene begins with TCG, which has been identified as the start codon for all *Bactrocera COI* genes. The 1004-bp A + T-rich region of *B. depressa* is the third longest, after *B. minax* and *B. scutellata*, of the *Bactrocera* species for which the whole mitogenome has been sequenced. Phylogenetic analysis using the 13 PCGs of *Bactrocera* species indicated that *B. depressa* is a sister to the sister group containing *B. tau* and *B. cucurbitae* with the highest nodal support (Bayesian posterior probability =1.0).

The pumpkin fruit fly, *B. depressa* (Diptera: Tephritidae), is an economically damaging pest of pumpkin and turban squash (Han et al. 1994; Kim et al. 1999). It is distributed in the mountainous regions of Korea, Japan, and Taiwan (Han et al. 1994; Kim et al. 1999); the females lay eggs inside young pumpkins, inside which the hatched feeding larvae cause severe damage (Yoshifumi 1952; Kim et al. 1999).

In this study, we sequenced the complete mitochondrial genome (mitogenome) of *B. depressa* to determine the mitogenomic characteristics of this species and its phylogenetic relationships within *Bactrocera*. Several adults were captured from a pumpkin farm located in Gunpo City, Gyunggi-do Province, South Korea (37°19′41′′N, 126°55′55.2′′E), in September 2015. A voucher specimen was deposited in Chonnam National University, Gwangju, Korea, under the accession number CNU5743.

By using the total DNA as a template, two long overlapping fragments (*COI–CytB* and *CytB–COI*) were amplified, and subsequently 26 short overlapping fragments were amplified using the two long fragments as templates. Primers for the long and short fragments were newly designed from several existing *Bactrocera* mitogenome sequences (Zhang et al. 2014, 2015; Choudhary et al. 2015; Yong et al. 2015, 2016).

The *B. depressa* mitogenome is 15,832 bp in length and includes the typical sets of genes (2 rRNAs, 22 tRNAs, and 13 protein-coding genes [PCGs]) and a major non-coding A + T-rich region (GenBank accession number KY131831). The mitogenome size is within the range found in *Bactrocera* – from 15,815 bp (*B. oleae*; Nardi et al. 2003) to 16,043 bp (*B. minax*; Zhang et al. 2014). The 1004-bp A + T-rich region of *B. depressa* is the third longest among those of the sequenced *Bactrocera*, after that in *B. minax* (1140 bp; Zhang et al. 2014) and *B. scutellata* (1011 bp; Unpublished, GenBank accession number KP722192). In *B. depressa*, the A + T-rich region contains two repeat sequence composed of 33 bp that are located at the beginning and end of the A + T-rich region, respectively (15,058–15,090 and 15,780–15,812 in the genome). The gene arrangement of *B. depressa* is identical to that of the ancestral insect order found in the majority of insects (Boore 1999).

The A/T content among genes and regions varies markedly in the *B. depressa* mitogenome: 81.8% in the A + T-rich region, 74.5% in *lrRNA*, 74.5% in tRNAs, 73.1% in *srRNA*, and 68.8% in PCGs. 12 of the *B. depressa* PCGs begin with a typical ATN start codon (six with ATG, four with ATT, one with ATC, and one with ATA), whereas the *COI* gene begins with the atypical TCG (Serine) sequence, as has been found in all sequenced *Bactrocera* mitogenomes (Zhang et al. 2014; Choudhary et al. 2015; Yong et al. 2015, 2016). The *B. depressa* mitogenome has a total of 128 bp of intergenic spacer sequences, spread over 14 locations, ranging in size from 1 to 38 bp. Among these, three locations have spacer sequences longer than 10 bp: 38 bp between *trnR* and *trnN*, 18 bp between *trnE* and *trnF*, and 15 bp between *trnS_2_* and *ND1*. These are composed of 79%, 83%, and 87% A/T nucleotides, respectively.

We performed a phylogenetic analysis of *Bactrocera* by using the 13 PCGs and two dipteran species belonging each to the same subgenus and superfamily of *Bactrocera* as outgroups considering a previous multigene fragment-based phylogeny of the dipteran Tribe Dacini (Krosch et al. 2012). The Bayesian inference method was performed using the GTR + GAMMA + I model in CIPRES Portal v. 3.1 (Miller et al. 2010). The results showed that *B. depressa* was placed as a sister to the sister group containing *B. tau* and *B. cucurbitae* with the highest nodal support (Bayesian posterior probability =1.0). Recent multigene fragment-based phylogenetic analysis of the Dacini has shown that the current subgeneric taxonomic designation of *Bactrocera* is not consistent with the phylogenetic clustering pattern (Krosch et al. 2012). Similarly, in our phylogenetic analysis, *B.* (*Paradacus*) *depressa*, which is classified as a member of the subgenus *Paradacus*, was grouped together with the sister group containing *B.* (*Zeugodacus*) *tau* and *B.* (*Zeugodacus*) *cucurbitae*, which are classified as the members of the subgenus *Zeugodacus* (Figure 1).

**Figure 1.**
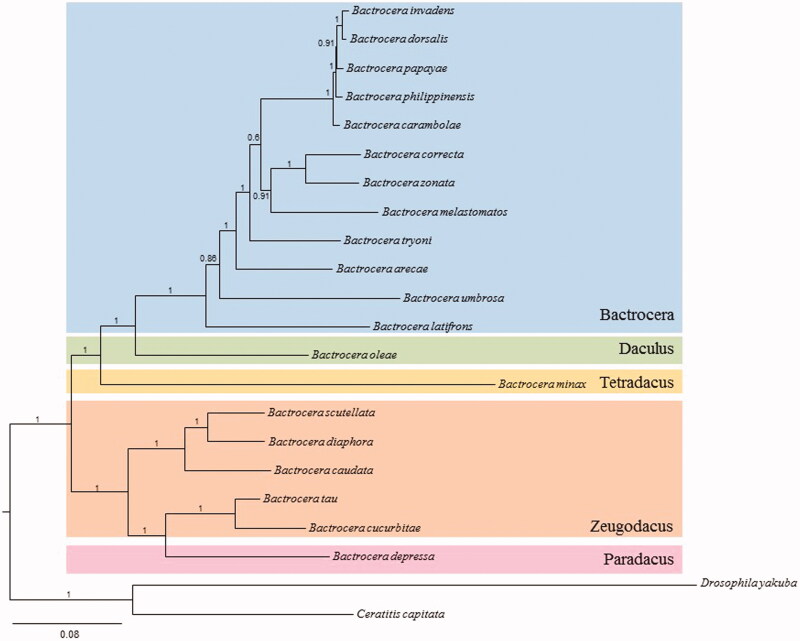
Phylogeny of *Bactrocera* based on 13 protein-coding genes, constructed using the Bayesian inference method. Values at each node are percentage Bayesian posterior probabilities. The scale bar indicates the number of substitutions per site. Subgeneric names are provided on the right side of the tree. The two dipteran species *D. yakuba* and *Ceratitis capitata* were included as outgroups. GenBank accession numbers are as follows: *B. zonata,* KP296150 (Choudhary et al. 2015); *B. arecae,* KR233259 (Yong et al. 2015); *B. correcta,* JX456552 (Unpublished); *B. dorsalis,* DQ845759 (Unpublished); *B. philippinensis,* DQ995281 (Unpublished); *B. carambolae,* EF014414 (Unpublished); *B. papayae,* DQ917578 (Unpublished); *B. tryoni,* HQ130030 (Nardi et al. 2010); *B. umbrosa,* KT881558 (Yong et al. 2016); *B. melastomatos,* KT881557 (Yong et al. 2016); *B. latifrons,* KT881556 (Yong et al. 2016); *B. invadens,* KX534207 (Zhang et al. 2016a); *B. diaphora,* KT159730 (Zhang et al. 2016b); *B. scutellata,* KP722192 (Unpublished); *B. tau,* KP711431 (Tan et al. 2016); *B. minax,* HM776033 (Zhang et al. 2014); *B. cucurbitae,* JN635562 (Unpublished); *B. caudata,* KT625492 (Yong et al. 2016); *B. oleae,* AY210703 (Nardi et al. 2003); *D. yakuba,* NC001322 (Clary & Wolstenholme 1985); and *C. capitata* AJ242872 (Spanos et al. 2000).
